# Temporal and demographic trends in cardiogenic shock and chronic ischemic heart disease-related mortality among U.S adults aged 45 years and older: a 25 year nationwide analysis with ARIMA forecasting

**DOI:** 10.1186/s12872-026-05859-w

**Published:** 2026-04-17

**Authors:** Faizan Ahmed, Muhammad Abdullah, Haris Bin Tahir, Bilal Qammar, Muhammad Shees Hunain, Haider Hussain Shah, Madeeha Shafqat, Shiraz Aslam, Taha Alam, Tehmasp Rehman Mirza, Dinesh Kumar, Abdul Waheed, Mohammad Amir Hossain, Mohammad Omar Butt, Fawaz Alenezi

**Affiliations:** 1 Jersey Shore University Medical Center, Neptune, NJ USA; 2 Shalamar Medical and Dental College, Lahore, Pakistan; 3 Lahore General Hospital, Lahore, Pakistan; 4 Sir Ganga Ram Hospital, Lahore, Pakistan; 5 Bayhealth Hospital, Kent Campus, Dover, DE USA; 6 Geisinger Medical Center, Danville, PA USA; 7 Ameer-ud-Din Medical College, Lahore, Pakistan; 8 Dow University of Health Sciences, Karachi, Pakistan; 9 Memorial Satilla Health, Waycross, GA USA; 10 Duke University School of Medicine, Durham, NC USA

**Keywords:** Cardiogenic Shock, Chronic Ischemic, Heart Disease, Mortality Trends, Age-Adjusted Mortality Rate, Health Disparities

## Abstract

**Background:**

Cardiogenic shock (CS) and chronic ischemic heart disease (CIHD) remain major contributors to cardiovascular mortality in the United States. Although mortality related to ischemic disease declined in the early 2000s, recent trends suggest a resurgence.

**Methods:**

This population-based study utilized the Centers for Disease Control and Prevention’s Wide-Ranging OnLine Data for Epidemiologic Research (CDC WONDER) database from 1999 to 2023. Deaths were identified using ICD-10 codes R57.0 (CS) and I25 (CIHD). Adults aged ≥ 45 years were included. Crude mortality rates (CMRs) and age-adjusted mortality rates (AAMRs) were calculated, with temporal changes assessed using Joinpoint regression to estimate Annual Percent Change (APC) and Average Annual Percent Change (AAPC).

**Results:**

Between 1999 and 2023, deaths attributed to CS and CIHD accounted for 78,903 cases among adults ≥ 45 years. Overall AAMR ranged from 35.88 per million in 1999 to 37.44 per million in 2023, with a downward trend from 1999 to 2012 followed by an upward trend thereafter. Males (51,080 deaths) exhibited rising mortality (AAPC: +0.50, *p* < 0.001), while females (27,823 deaths) showed a decline (AAPC: − 0.91, *p* < 0.0004). Non-Hispanic Black adults demonstrated the steepest increase (AAPC: +1.85, *p* < 0.001). The burden was greatest in adults ≥ 65 years, but the 45–64 group showed the fastest rise (AAPC: +1.67, *p* < 0.001).

**Conclusion:**

Mortality from cardiogenic shock and chronic ischemic heart disease among U.S. adults ≥ 45 years declined initially but reversed after 2012. Rising mortality in middle-aged adults, persistent racial disparities, and geographic inequities highlight urgent gaps in prevention and acute care.

**Supplementary Information:**

The online version contains supplementary material available at 10.1186/s12872-026-05859-w.

## Introduction

Chronic Ischemic Heart Disease (IHD), also known as Coronary Artery Disease (CAD), is characterized by restricted myocardial blood flow due to narrowing or obstruction of the coronary arteries [1]. The condition is primarily caused by atherosclerosis [2] and, less commonly, by coronary vasospasm. Globally, CAD remains a major public health challenge, contributing to approximately 32% of all deaths in 2019 [3].

Cardiogenic shock (CS) is a severe and life-threatening clinical state resulting from inadequate tissue perfusion secondary to critically reduced cardiac output [4]. Among patients with acute myocardial infarction (AMI), CS is associated with an alarmingly high in-hospital mortality rate of up to 50% [5]. Although arrhythmias and chronic heart failure can precipitate CS, left ventricular dysfunction secondary to IHD remains the predominant underlying cause [6].

Mortality trends have been extensively examined for cardiogenic shock (CS) and ischemic heart disease (IHD) independently. Aside from a temporary increase between 2019 and 2020, IHD-related mortality has shown a consistent decline across major U.S. demographics during the 22-year period from 1999 to 2020. In contrast, the age-adjusted mortality rate (AAMR) for heart failure–related CS increased significantly between 2009 and 2021. Notably, both retrospective studies reported poorer outcomes in rural populations, suggesting the influence of shared risk factors contributing to morbidity and mortality in both IHD and CS.

Moreover, with an aging and increasingly obese U.S. population, the disease burden of IHD remains substantial, accounting for 371,506 deaths in 2022 [7]. Given that atherosclerosis, the fundamental pathological mechanism underlying IHD [8], is also implicated in life-threatening CS, there is a critical need to examine concomitant mortality trends in these two closely related conditions.

Given the close pathophysiological relationship between chronic ischemic heart disease and cardiogenic shock, where ischemic myocardial injury remains a leading precipitant of shock, examining these conditions in combination allows for a more integrated assessment of ischemic disease burden and its most severe clinical manifestation. This approach enables the evaluation of mortality patterns across a continuum of disease progression, from chronic ischemia to acute hemodynamic collapse.

Additionally, this combined analysis could help elucidate long-term associations between IHD and CS, potentially informing prevention and management strategies. Addressing this knowledge gap may provide valuable insight into demographic and clinical variations in mortality among patients with concurrent IHD and CS, paving the way for more targeted investigations into the underlying mechanisms driving these cardiovascular outcomes.

## Methodology

### Study setting

This investigation utilized mortality data obtained from the Centers for Disease Control and Prevention’s Wide-ranging Online Data for Epidemiologic Research (CDC WONDER) database, which compiles information from U.S. death certificates. The study examined deaths attributed to Cardiogenic Shock and Chronic Ischemic Heart Disease occurring between 1999 and 2023. Case identification was performed using International Classification of Diseases, Tenth Revision (ICD-10) codes “R57.0” for cardiogenic shock and “I25”, which includes atherosclerotic cardiovascular disease, myocardial infarction, coronary artery aneurysm, and other chronic ischemic heart disease manifestations. The database contains cause-of-death information from all 50 states and the District of Columbia. Records from the Multiple Cause of Death Public Use dataset were analyzed to identify eligible cases. Adults were defined as individuals aged 45 years and older at the time of death. Institutional review board approval was not required because the study used publicly available, deidentified government data. Reporting followed the Strengthening the Reporting of Observational Studies in Epidemiology (STROBE) guidelines. The general methodological framework for mortality data extraction and rate calculations has been described previously and is summarized here for clarity [9].

### Data extraction

Information obtained from death certificates included population counts, year of death, place of death, and demographic variables such as sex, age, race, and ethnicity. Geographic variables included regional classification, state-level data, and urban–rural designation. Deaths were recorded across multiple settings, including hospitals, private residences, hospice facilities, nursing homes, and long-term care institutions. Urban–rural status was assigned according to the National Center for Health Statistics Urban–Rural Classification Scheme. Urban areas comprised large metropolitan regions with populations of 1 million or more and medium or small metropolitan areas with populations between 50,000 and 999,999. Rural areas were defined according to the 2013 U.S. Census criteria as regions with fewer than 50,000 residents, along with additional qualifying counties. Geographic regions were categorized into the Northeast, Midwest, South, and West in accordance with U.S. Census Bureau definitions.

### Statistical analysis

Annual crude mortality rates (CMRs) and age-adjusted mortality rates (AAMRs) were calculated for each year of the study period. CMRs were determined by dividing the total number of deaths in a given year by the corresponding population. AAMRs were computed using the direct standardization method and adjusted to the 2000 U.S. standard population to account for differences in age distribution over time. Trends in CMRs and AAMRs were evaluated using the Joinpoint Regression Program to estimate Annual Percent Change (APC) and Average Annual Percent Change (AAPC) with 95% confidence intervals. Statistical significance was defined as a p-value less than 0.05. Time-series forecasting of mortality trends was performed using autoregressive integrated moving average (ARIMA) models implemented in Python version 3.13.2 with the statsmodels package. Model parameters (p, d, q) were assessed iteratively, with the search range for each parameter increased from orders 0–3 to 0–5 to ensure a more robust model selection; the optimal model was selected based on the lowest root mean square error (RMSE) during validation. Prediction intervals were assessed for plausibility, and model specifications were iteratively refined to ensure clinically interpretable (non-negative) estimates.

## Results

### Annual trends

**Fig. 1:**
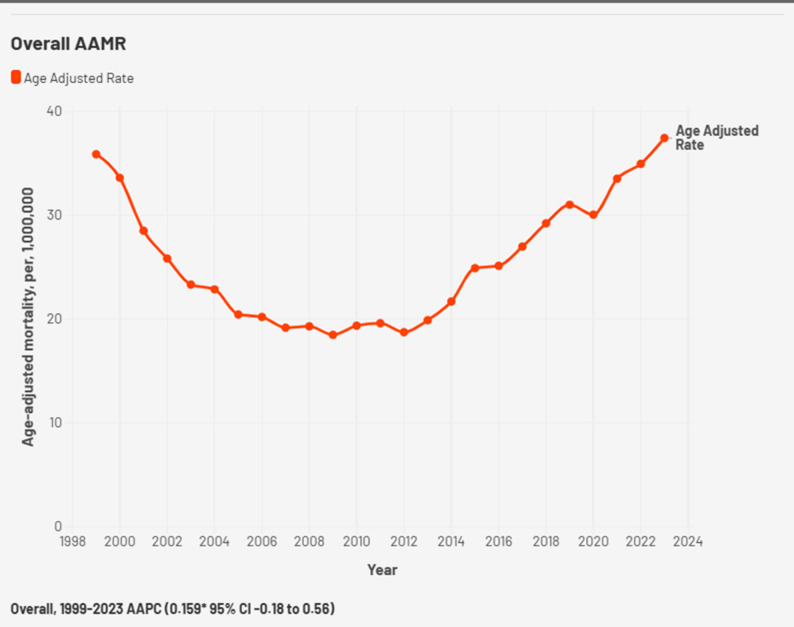
Overall cardiogenic shock and chronic ischemic heart disease -related AAMR per 1,000,000 in the United States, 1999 to 2023

Cardiogenic Shock and Chronic Ischemic Heart Disease caused a total of 78,903 deaths in the US from 1999 to 2023 (Supplemental Table 1). The age-adjusted mortality rates (AAMR) ranged between 35.88 (95% CI 34.68–37.09) in 1999 and 37.44 per million (95% CI 36.46–38.42) in 2023 (Fig. 1, Supplemental Table 2). The highest AAMR was reported in 2023 at 37.44 per million. The rates had demonstrated a downward slope in the years 1999–2012 followed by an upward trend from 2012 to 2023 indicating significant changes in AAMR in these eras. The greatest decrease in AAMR was observed from 1999 to 2005 (APC: -9.20*, 95% CI: -13.50 to -6.05, *p* < 0.002). The largest numerical increase in AAMR was observed from 2012 to 2015 (APC: 9.26, 95% CI: -1.91 to 12.61), although this change did not reach statistical significance. The largest statistically significant increase occurred from 2015 to 2023 (APC: 5.41*, 95% CI: 1.88 to 6.76, *p* < 0.05) (Supplemental Table 3).

### Gender

**Fig. 2:**
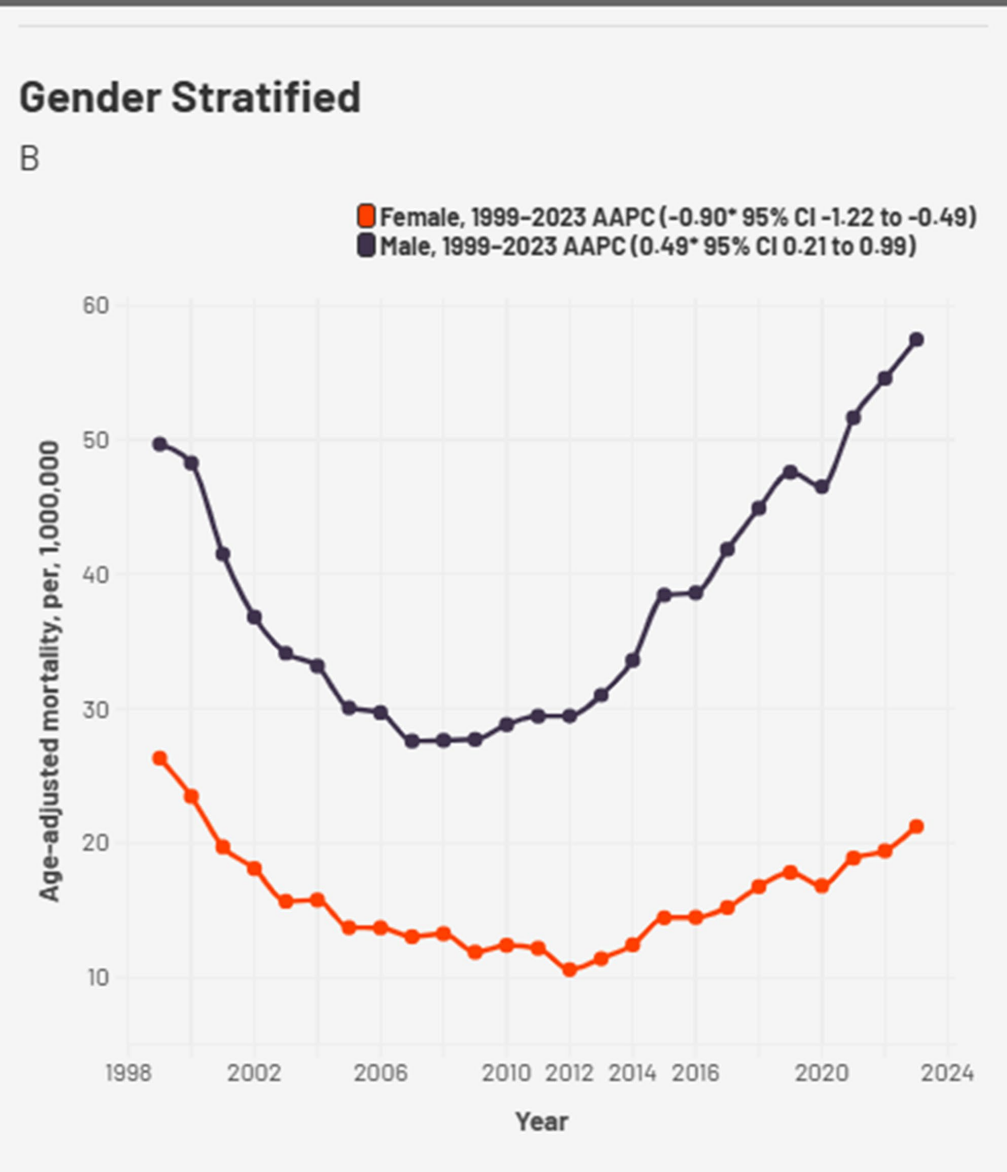
Sex-stratified cardiogenic shock and chronic ischemic heart disease -related AAMRs per 1,000,000 in the United States, 1999 to 2023

Segregation of the database by gender revealed a greater burden in AAMR for Males throughout the time period though trends were similar in males and females. Males had a total of 51,080 deaths and the females had a total of 27,823 deaths from 1999 to 2023, respectively. The AAMR for females fell from 26.33 in 1999 to 21.26 per million in 2023 with an AAPC of -0.907 (95% CI -1.22 to -0.498, *p* < 0.0004). The AAMR for males rose from 49.68 in 1999 to 57.45 per million in 2023 with an AAPC of 0.498 (95% CI 0.21 to 0.99, *p* < 0.001) (Fig. 2, Supplemental Table 2). APC trends for both genders were consistent from 1999 to 2023 (Supplemental Table 3).

### Race

**Fig. 3:**
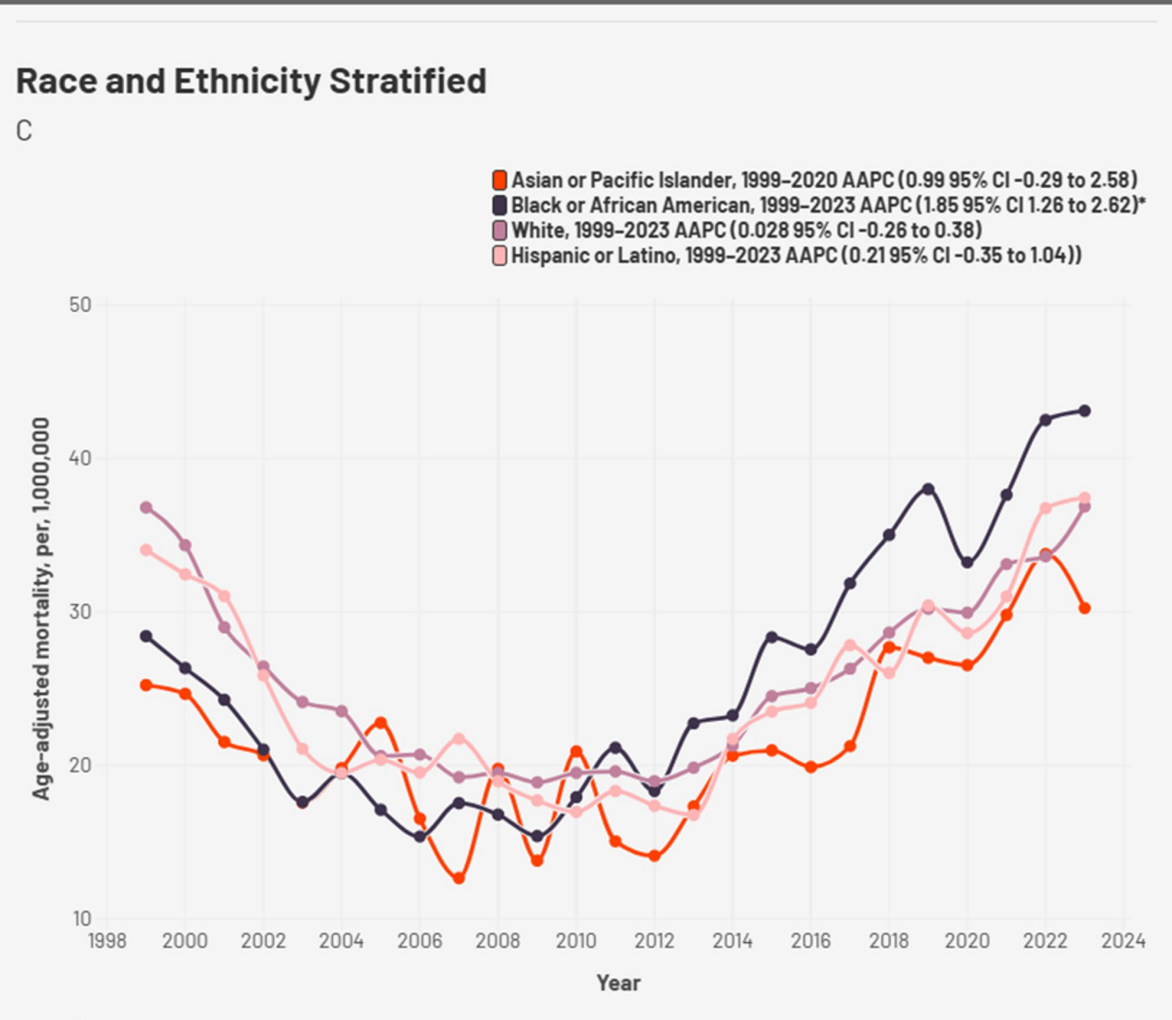
Race/ ethnicity stratified cardiogenic shock and chronic ischemic heart disease -related AAMR per 1,000,000 deaths in the United States, 1999 to 2023

Racial trends showed a drastically higher trend in Non-Hispanic (NH) American Indian individuals compared to other races with an eventual AAMR of 53.04 per million in 2023. Though initially unreliable, NH American Indians had the highest peak of the other races with an AAMR of 53.04 in 2023; followed by NH Blacks (43.12 in 2023), Hispanics (37.45 in 2023) and NH White (36.88 in 2023) (Fig. 3, Supplemental Table 4). Interestingly, rise in AAMR was steepest in NH Black (AAPC: 1.85* 95% CI 1.26 to 2.62, *p* < 0.001) owing to a period of sustained rise from 2006 to 2023 (APC: 6.68, *p* < 0.001) (Supplemental Table 3). Unlike other races, which had a sustained rise from 2012 till 2023. A downward trend was observed in all races till 2012 excluding NH black.

### Age groups

Age wise distribution was starkly skewed towards adults older than 65 years. Stratification revealed 15,367 deaths (19.4% of total deaths) in adults aged 45–64 years while 63,536 deaths occurred in adults aged above 65. Regardless, 45–64 age groups have AAMR on a rising trend from 2010 onwards. (APC: 9.25, 95% CI 8.35–11.47, *p* < 0.001) from 2010 to 2018 and (APC: 4.42, 95% CI 2.63–5.68, *p* < 0.001) from 2018 to 2023. AAMR in adults 45–64 increased from 8.01 to 12.04 from 1999 to 2023 (AAPC: 1.67, 95% CI 1.42 to 1.94, *p* < 0.001). On the other hand, AAMR in adults aged above 65 fell from 84.86 in 1999 to 82.08 in 2023 (AAPC: -0.12, 95% CI -0.39 to 0.19, *p* < 0.378).

### Geographic trends (census and states)

**Fig. 4:**
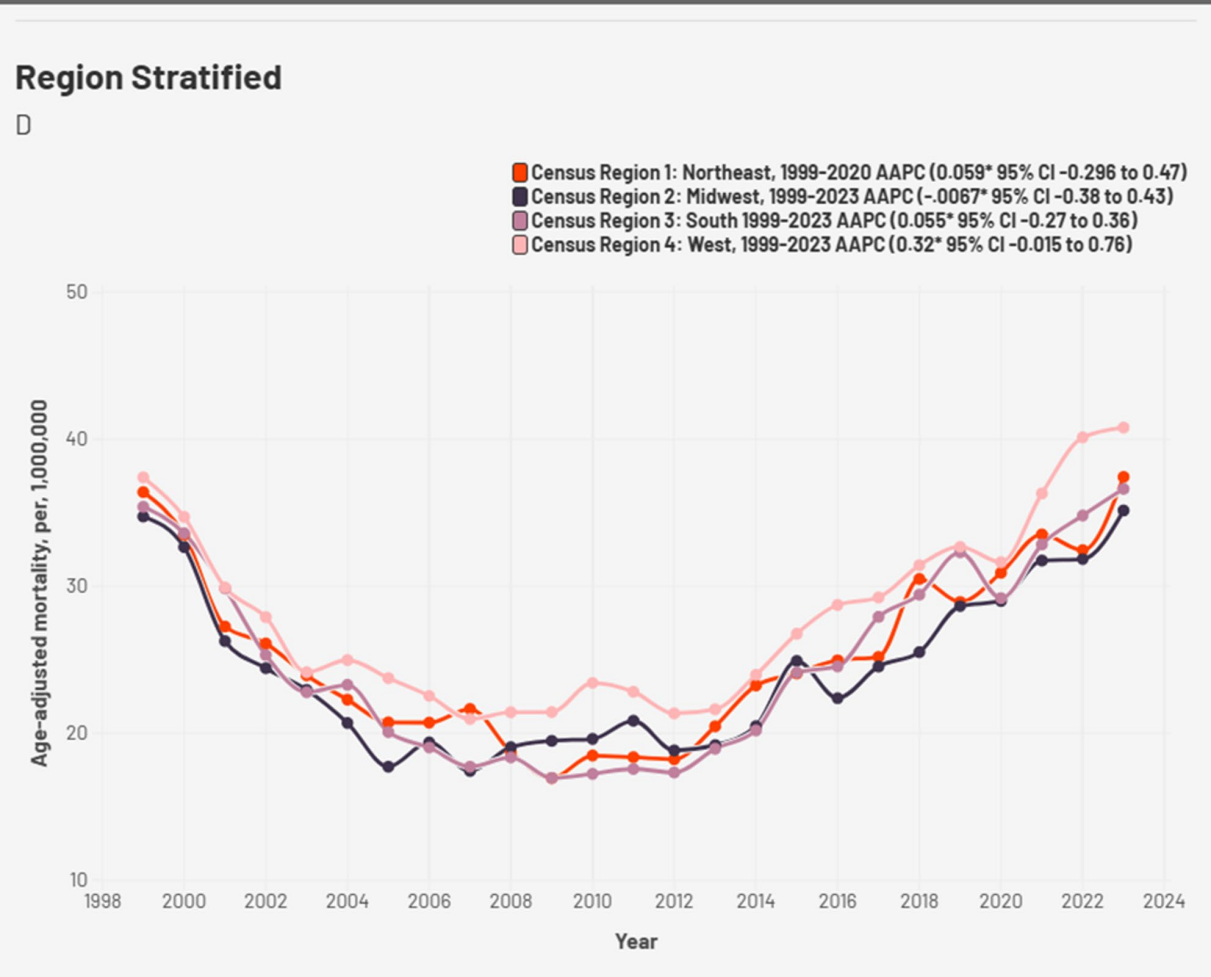
Cardiogenic shock and chronic ischemic heart disease -related AAMR per 1,000,000 Stratified by regions in the United States, 1999 to 2023

The AAMR across the four census regions were generally similar with a slightly higher burden in the West while the Midwest had a comparatively lower burden. Highest AAMR in the West was 40.8 per million in 2023, Northeast (37.44 in 2023) followed by South (36.63 in 2023), and Midwest (35.16 in 2023) (Fig. 4, Supplemental Table 5). The strongest rise in AAMR was in the Southern region (2012–2017 APC: 10.3, *p* < 0.002), followed by the Western region (2012–2023 APC: 6.03, *p* < 0.001).

**Fig. 5:**
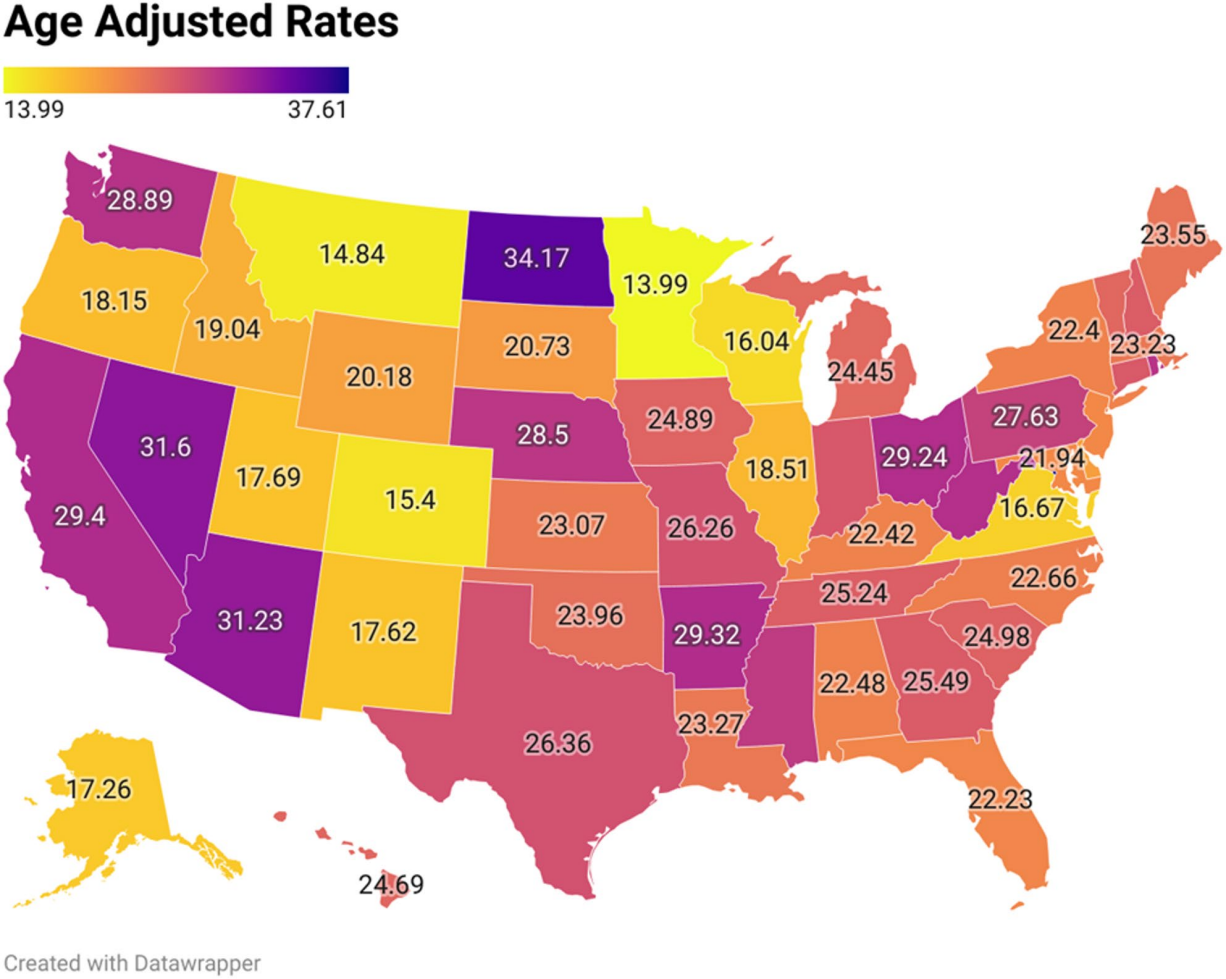
State-stratified cardiogenic shock and chronic ischemic heart disease -related AAMRs per 1,000,000 in United States 1999–2020

State wise analysis occurred over two periods 1999 to 2020 and 2021 to 2023. Both periods yielded varied results with Arkansas, California, Arizona, Nevada, District of Columbia for the first period and Pennsylvania, Tennessee, West Virginia, Mississippi, Washington and Nevada for the second period in top 90th percentile. For the 10th percentile, Alaska, Virginia, Wisconsin, Colorado, Montana, and Minnesota were noted in the first period. In the second period, Connecticut, Virginia, Montana, Maine, Delaware, and Utah were found in the 10th percentile range (Fig. 5, Supplemental Tables 6,7).

### Urbanization

**Fig. 6:**
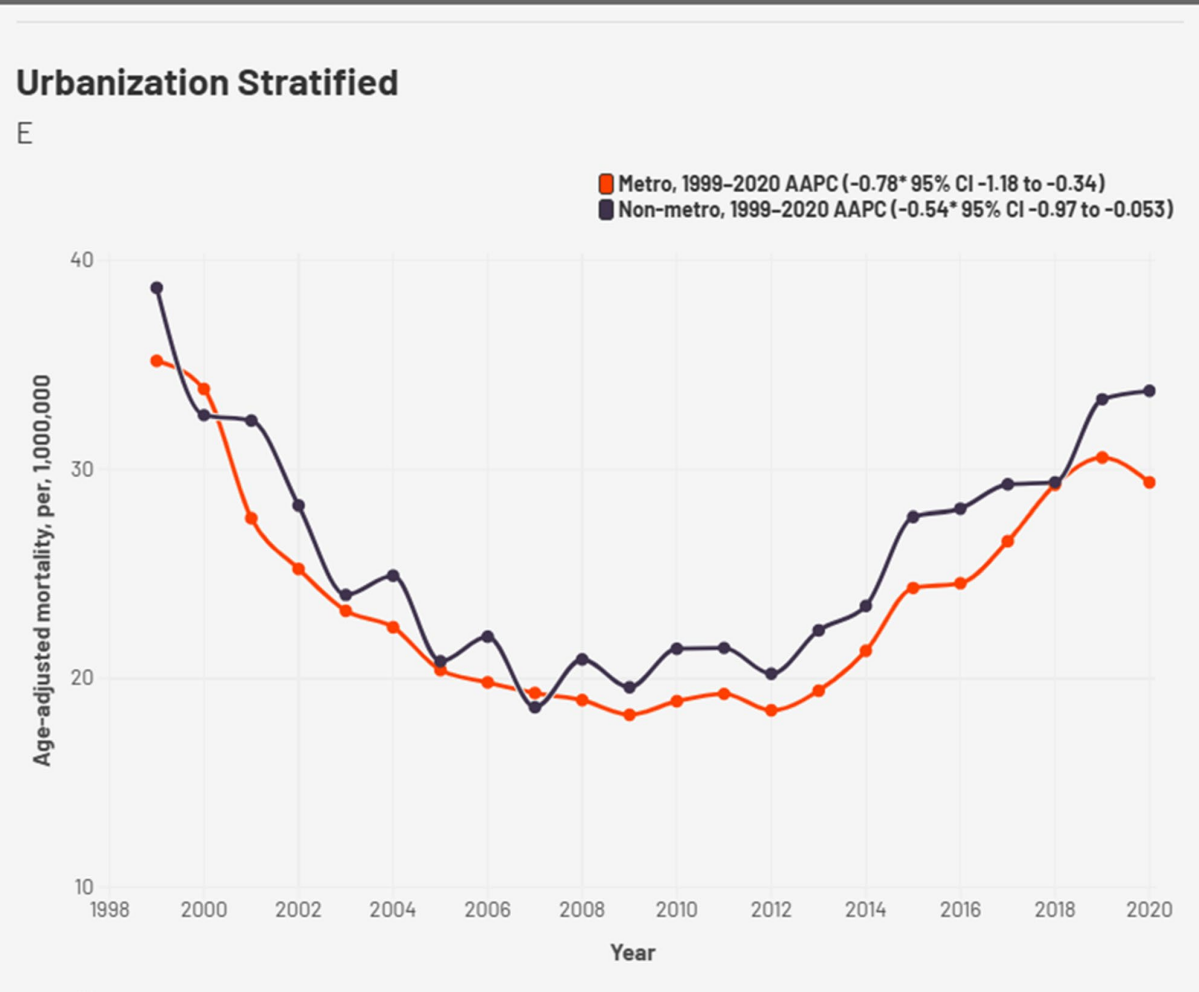
Urbanization stratified cardiogenic shock and chronic ischemic heart disease -related AAMR per 1,000,000 in the United States, 1999 to 2020

Stratification by urbanization up to 2020 revealed 81% of deaths occurred in metropolitan areas. The AAMR in non-metro was relatively higher throughout. Both regions faced a decline in AAMR initially till 2012 for metro and 2007 for non metro followed by a drastic increase after that (Fig. 6, Supplemental Table 8). The APC from 2012 to 2018 for metro areas was 8.07, *p* < 0.068 and 4.75, *p* < 0.001 for non-metro from 2007 to 2020 (Supplemental Table 3). AAMR peaked for both in 1999 at 35.19 for metro and 38.69 for nonmetro.

### Place of death

**Fig. 7:**
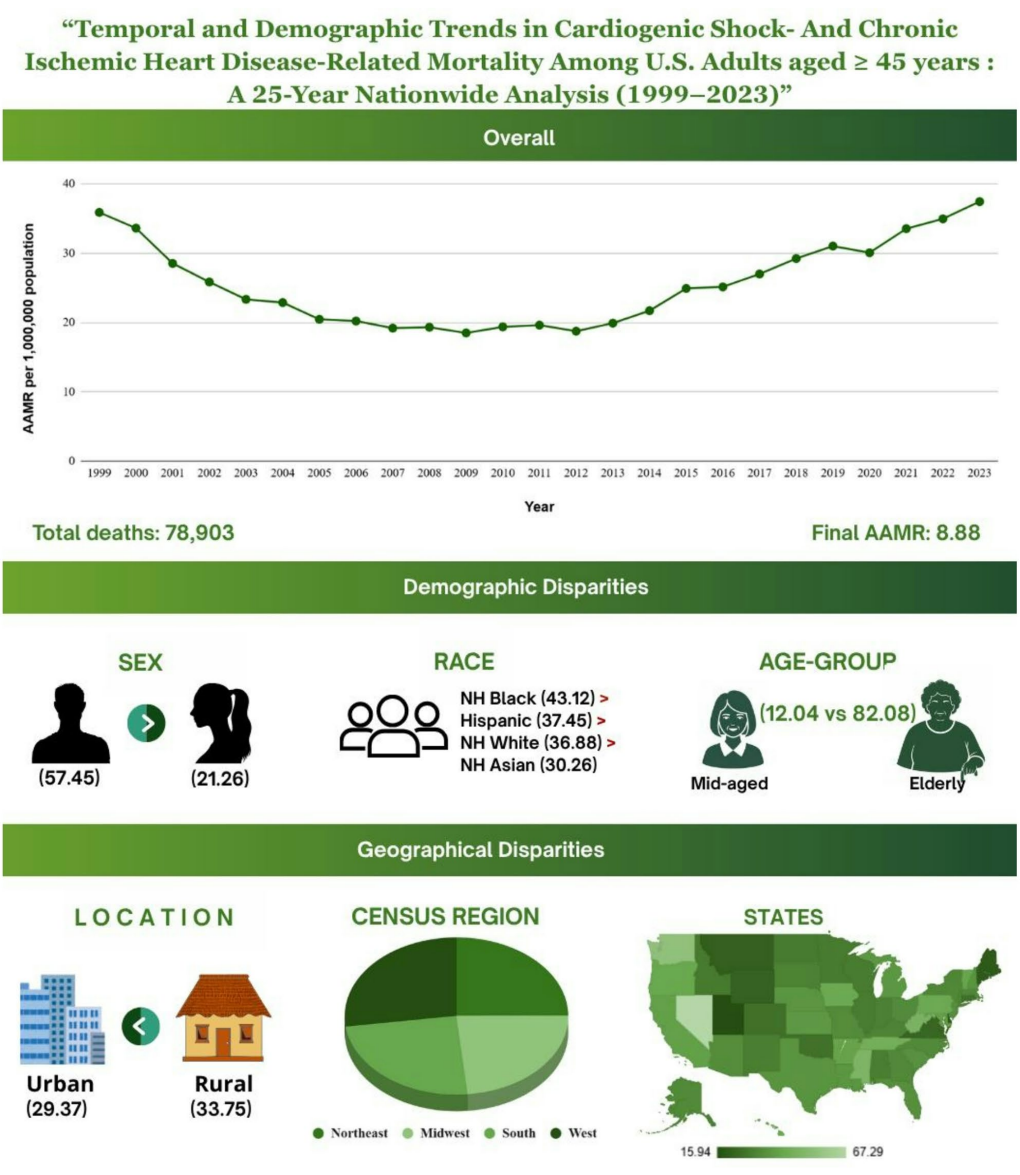
Central illustration: demographic profiles in cardiogenic shock and chronic ischemic heart disease -related mortality among adults 45 to 85 + In the United States, 1999 to 2023

Inpatient Facility recorded the highest number of deaths at 52,738 (84%), followed Outpatient or ER (3063; 4.88%), Nursing Home (2980; 4.75%), Decadent’s Home (2650, 4.2%), Other (475, 0.75%) respectively (Supplemental Table 9). A central illustration (Fig. 7) shows the demographic profiles of cardiogenic shock and chronic ischemic heart disease–related mortality among adults aged 45 to 85 + years in the United States from 1999 to 2023.

### Forecast

**Fig. 8:**
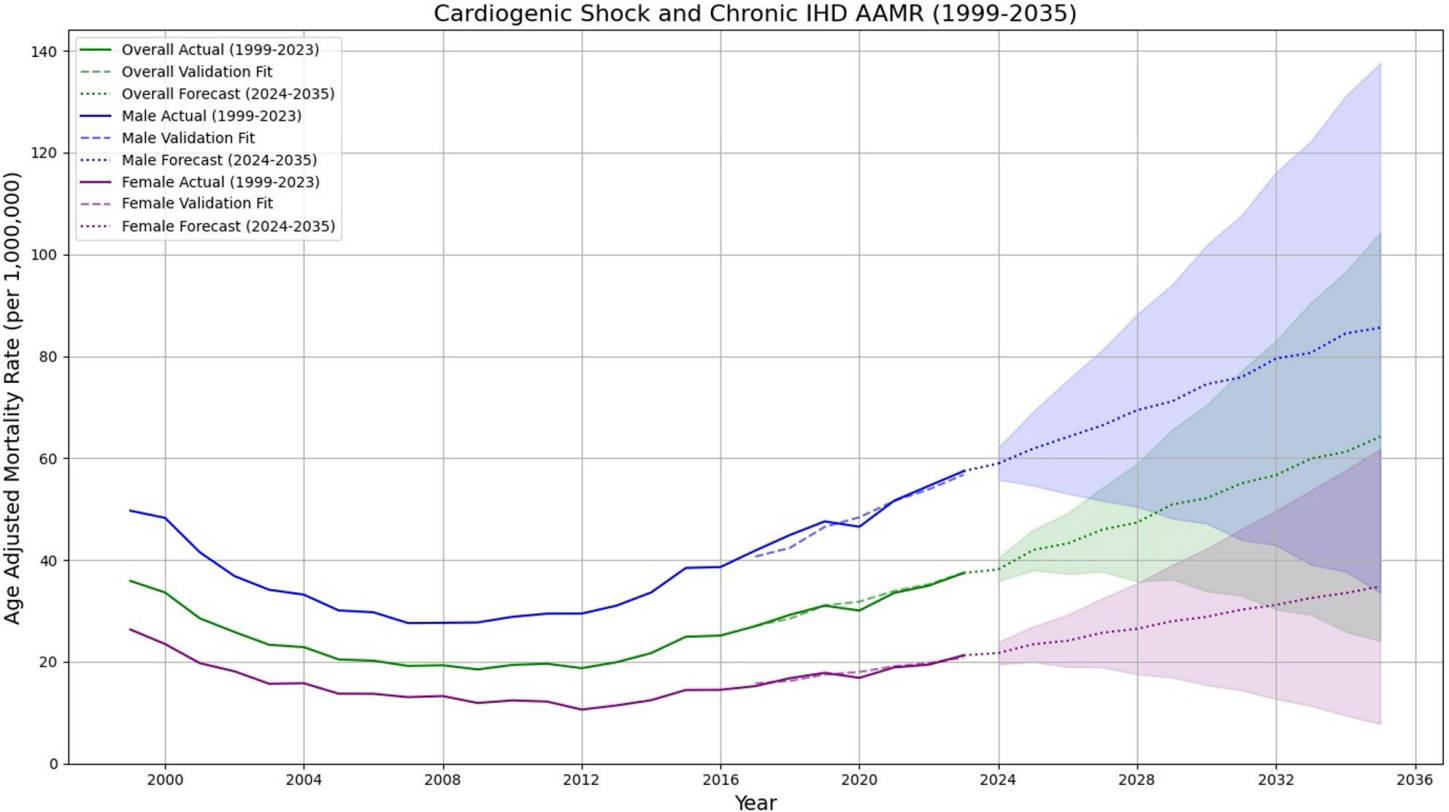
Time-series forecasting of overall and gender-stratified mortality rates using ARIMA modeling (2024–2035)

Forecasting database trends through 2035 suggests rising mortality, with the overall AAMR estimated to reach 64.23 in 2035 (95% prediction interval: 24.11–104.4). Gender-stratified projections show a similar upward trajectory; AAMRs are projected to reach 85.58 per million (95% prediction interval: 33.56–137.6) for males and 34.80 (95% prediction interval: 7.79–61.81) for females. The projected average annual change in mortality is estimated at 4.87 overall, with increases of 3.59 in males and 4.46 in females over the forecast period. These projections represent modeled estimates and should be interpreted as exploratory rather than inferential findings (Fig. 8).

## Discussion

This comprehensive analysis of national mortality data from 1999 to 2023 highlights evolving patterns in cardiogenic shock (CS) and chronic ischemic heart disease (CIHD) among U.S. adults aged ≥ 45 years. On the whole, although the mortality rate has decreased in the early 2000s, the current turnaround of the situation since 2012 supports the notion of the ongoing difficulties in cardiovascular care and prevention [6]. Of particular concern is the noted increase in the range of revascularization, pharmacotherapy, and mechanical circulatory support (MCS), implying that the acute management improvement has not been wholly translated into long-term mortality [10]. The biphasic tendency that registered sharp mortality decreases between 1999 and 2012 and then a rising tendency until 2023 is consistent with macro tendencies in the epidemiology of ischemic heart disease. The Early declines are more likely to be a result of adoption of guideline-based therapies, better management of the acute coronary syndrome (ACS) and secondary prevention measures [11].

Nevertheless, the reversal observed after 2012 may, in part, be attributed to a rising prevalence of cardiometabolic comorbidities, including diabetes, obesity, and chronic kidney disease, which are known to adversely impact cardiovascular outcomes. However, this trend is likely multifactorial. Changes in population demographics, including an aging population and improved survival with chronic cardiovascular conditions, may have contributed to a higher burden of advanced disease. Additionally, the growing prevalence of heart failure and evolving epidemiology of myocardial infarction may have influenced the incidence and severity of cardiogenic shock. Disparities in access to timely revascularization and advanced cardiac care, along with regional variations in healthcare infrastructure, may also play a role. More recently, disruptions in healthcare delivery and delayed presentations during periods such as the COVID-19 pandemic may have further exacerbated adverse outcomes [12, 13]. Collectively, these factors highlight the complex and multifactorial drivers underlying the observed mortality trends.

The gender difference observed in this analysis is notable. Male AAMRs increased over time, whereas female AAMRs declined. Prior literature suggests that men tend to develop ischemic disease at an earlier age and often carry a higher burden of risk factors, including smoking and metabolic syndrome [14]. Women, on the other hand, have historically been underdiagnosed and undertreated. The observed improvement in female mortality may reflect a combination of factors, including increased awareness, improved risk factor control, and public health initiatives; however, these explanations remain speculative and warrant further investigation. These divergent trends underscore the importance of gender-specific approaches, particularly targeting younger and middle-aged men [15].

Racial differences are a major result. The sustained increase in mortality has increased steadily among Non-Hispanic Black adults since 2006, even though the general trend in the mortality of cardiovascular disease has improved nationwide. Likely causes are structural inequities, such as decreased access to reperfusion therapies, decreased rates of advanced heart failure interventions, and socioeconomic disadvantage [16]. There was also an imbalance of the mortality of non-Hispanic American Indians, which was reported before when it was already stated that this population was at a higher risk of cardiovascular diseases [17]. The results support the relevance of equity-oriented policies, community-based prevention strategies, and better access to high-acuity cardiac care in underserved groups. Adults over the age of 65 continued to represent the majority of deaths, and the most alarming pattern was increased AAMR in the age group of 45–64 years [18]. This is an indication that premature mortality is emerging as something more of a load and possibly an earlier onset of cardiometabolic disease, inactive lifestyles, and loopholes in identifying primary prevention. The increase in younger adults can annul the gains made in older groups, and this underscores the urgency of intensive preventive efforts against modifiable risk factors in midlife [19].

Our findings align with those of Naveed et al. (2025), who reported rising cardiogenic shock mortality among heart failure patients aged ≥ 25 years, using the same CDC WONDER database. Their study demonstrated a nearly fourfold increase in AAMRs from 1.2 to 4.6 per 100,000 between 1999 and 2023, with particularly steep rises from 2009 to 2021 (APC: 14.17) and continued increases thereafter [20]. Both analyses highlight higher mortality in men, Black adults, and rural populations. However, important distinctions exist. Whereas Naveed et al. focused exclusively on CS in the context of heart failure, our study combined CS and CIHD mortality among older adults, yielding a broader perspective on ischemic burden and related shock syndromes [21]. Their younger inclusion threshold (≥ 25 years) captured additional premature mortality not addressed in our ≥ 45-year analysis, possibly explaining their steeper upward trajectory. In contrast, our data reveal stabilization or decline in ≥ 65-year adults, coupled with alarming increases in 45–64-year groups, which may represent a narrowing overlap with their younger cohort [22]. Taken together, both studies underscore that CS mortality is rising across populations but that etiology (HF vs. CIHD) and age at onset shape distinct epidemiologic trajectories.

Geographic heterogeneity was evident, with the South and West regions showing the steepest increases in mortality. These patterns align with regional disparities in obesity, diabetes prevalence, and healthcare access. These regional differences may also reflect underlying healthcare system factors, including disparities in access to specialized cardiovascular centers, rural hospital closures, and variations in preventive care delivery across regions. Furthermore, non-metropolitan areas consistently exhibited higher AAMRs, underscoring challenges in timely access to advanced cardiovascular services, limited hospital resources, and fewer specialized shock centers. State-level differences further emphasize that mortality is shaped not only by biology but also by healthcare infrastructure, policy environments, and social determinants of health [23]. The predominance of inpatient deaths (84%) highlights the acute and severe nature of CS and CIHD-related mortality. However, notable proportions of deaths occurring in nursing homes, private residences, and emergency departments suggest that some patients either did not access advanced hospital care in time or were managed conservatively due to comorbidities, frailty, or limitations of care. This pattern raises questions about end-of-life planning and the adequacy of palliative approaches for advanced cardiac disease. Looking forward, projection models suggest that unless risk factor trends are reversed, the burden of CS and CIHD mortality will likely increase by 2035, particularly in younger adults and high-risk racial groups. These forecasts should be interpreted as exploratory projections rather than inferential estimates, as they are derived from modelled data rather than observed outcomes.

Public health initiatives must emphasize early detection of ischemic heart disease, widespread adoption of preventive strategies, and equitable access to high-acuity cardiovascular care. Additionally, expansion of multidisciplinary shock teams and better integration of MCS may help reduce inpatient mortality. Research should also address long-term survivorship and quality of life among CS survivors, as survival without functional recovery may not represent true success. Emerging technologies, including artificial intelligence–guided triage and precision-based cardiovascular therapeutics, may also offer future solutions if integrated equitably. Preventive cardiology, telemedicine, and community-level interventions will be critical in bridging urban-rural and state-level divides.

This study’s strengths include its use of a comprehensive, nationwide database spanning nearly 25 years and standardized mortality coding that allows for consistent trend analysis. However, several limitations must be acknowledged. Death certificate data may misclassify underlying causes, particularly for cardiogenic shock, which is often recorded as a terminal event rather than a distinct diagnosis. Additionally, the analysis cannot capture granular clinical variables such as comorbidities, treatment strategies, or socioeconomic determinants, which may influence outcomes. Furthermore, although cardiogenic shock and chronic ischemic heart disease are closely related, they represent distinct clinical entities with differing epidemiological and therapeutic profiles; their combined analysis may limit the ability to attribute mortality trends to specific disease processes. Moreover, temporal associations observed in this study do not imply causality. Finally, projection models, while statistically robust, are inherently dependent on historical trends and may not fully account for future changes in healthcare delivery, risk factor prevalence, or therapeutic advancements. Forecast estimates should therefore be interpreted as exploratory projections, and the relatively wide prediction intervals reflect increasing uncertainty over longer forecast horizons.

## Conclusion

It is concluded that cardiogenic shock and chronic ischemic heart disease remain major contributors to mortality among U.S. adults aged ≥ 45 years, with 78,903 deaths recorded between 1999 and 2023. While overall mortality initially declined in the early 2000s, the reversal of this trend after 2012 reflects shifting cardiovascular epidemiology, rising comorbidities, and persistent inequities in access to advanced care. Male sex, non-Hispanic Black race, younger age groups (45–64 years), and residence in rural or high-burden states emerged as the most vulnerable categories, underscoring the uneven distribution of disease burden. When placed in the context of recent literature, such as the study by Naveed et al. on cardiogenic shock mortality in heart failure patients, our findings both corroborate and extend existing evidence. Their analysis demonstrated a nearly fourfold rise in shock-related mortality among adults ≥ 25 years, particularly in younger groups and disadvantaged populations. Our study confirms similar demographic disparities but highlights that the burden in older adults is increasingly driven by chronic ischemic heart disease, with concerning upward trends in the 45–64 age group. Together, these complementary perspectives emphasize that premature ischemic disease and shock syndromes are converging public health threats requiring urgent preventive and therapeutic interventions.

## Supplementary Information

Supplementary Material 1.

## Data Availability

All data used in this study are publicly available through the Centers for Disease Control and Prevention Wide-ranging Online Data for Epidemiologic Research (CDC WONDER) database. Data were accessed in accordance with the CDC’s data use guidelines.

## Abbreviations

AAMR: Age-Adjusted Mortality Rate
AAPC: Average Annual Percent Change
ACS: Acute Coronary Syndrome
AMI: Acute Myocardial Infarction
APC: Annual Percent Change
ARIMA: Autoregressive Integrated Moving Average
CAD: Coronary Artery Disease
CDC: Centers for Disease Control and Prevention
CI: Confidence Interval
CIHD: Chronic Ischemic Heart Disease
CMR: Crude Mortality Rate
CS: Cardiogenic Shock
ER: Emergency Room
ICD-10: International Classification of Diseases, Tenth Revision
IHD: Ischemic Heart Disease
MCS: Mechanical Circulatory Support
NH: Non-Hispanic
RMSE: Root Mean Square Error
STROBE: Strengthening the Reporting of Observational Studies in Epidemiology
WONDER: Wide-ranging Online Data for Epidemiologic Research
