# Supplementary material for: Temporal and demographic trends in cardiogenic shock and chronic ischemic heart disease-related mortality among U.S adults aged 45 years and older: a 25 year nationwide analysis with ARIMA forecasting
Source: BMC Cardiovasc Disord. 2026 Apr 17;26:466. doi: 10.1186/s12872-026-05859-w (PMC13231575; doi:10.1186/s12872-026-05859-w)
Supplement: Supplementary file 1 — Supplementary Material 1. [file 12872_2026_5859_MOESM1_ESM.docx]

**Supplementary Data**

**Supplemental Table 1 Number of Cardiogenic Shock and Chronic Ischemic Heart Disease-Related Deaths, Stratified by Sex and Race in Adults in the United States 1999-2023.**

| **Year** | **Overall** | **Women** | **Men** | **NH White** | **NH Black or African American** | **NH Asian or Pacific Islander** | **NH American Indian or Alaska Native** | **Hispanic or Latino** | **Population** |
| --- | --- | --- | --- | --- | --- | --- | --- | --- | --- |
| **1999** | 3413 | 1528 | 1885 | 2960 | 224 | 55 | 15 | 153 | 95153686 |
| **2000** | 3235 | 1378 | 1857 | 2787 | 213 | 60 | 13 | 151 | 96944389 |
| **2001** | 2792 | 1159 | 1633 | 2372 | 197 | 54 | Suppressed | 154 | 99781854 |
| **2002** | 2567 | 1084 | 1483 | 2192 | 174 | 50 | Suppressed | 136 | 102217733 |
| **2003** | 2358 | 952 | 1406 | 2026 | 151 | 50 | Suppressed | 119 | 104692428 |
| **2004** | 2345 | 962 | 1383 | 1994 | 167 | 55 | Suppressed | 114 | 107138553 |
| **2005** | 2141 | 857 | 1284 | 1777 | 150 | 74 | 12 | 126 | 109787199 |
| **2006** | 2152 | 858 | 1294 | 1805 | 145 | 53 | 18 | 126 | 112380379 |
| **2007** | 2083 | 840 | 1243 | 1704 | 166 | 45 | 13 | 151 | 114894084 |
| **2008** | 2144 | 861 | 1283 | 1751 | 169 | 72 | 11 | 137 | 117395131 |
| **2009** | 2100 | 790 | 1310 | 1732 | 152 | 56 | 19 | 136 | 119895863 |
| **2010** | 2229 | 836 | 1393 | 1798 | 184 | 85 | 19 | 140 | 121757429 |
| **2011** | 2332 | 837 | 1495 | 1849 | 234 | 68 | 14 | 164 | 124174484 |
| **2012** | 2297 | 752 | 1545 | 1832 | 210 | 71 | 15 | 163 | 126000296 |
| **2013** | 2490 | 823 | 1667 | 1948 | 265 | 90 | 15 | 166 | 127788037 |
| **2014** | 2785 | 912 | 1873 | 2126 | 287 | 115 | 23 | 228 | 129779643 |
| **2015** | 3276 | 1061 | 2215 | 2477 | 365 | 123 | 40 | 263 | 131826832 |
| **2016** | 3395 | 1098 | 2297 | 2579 | 362 | 125 | 30 | 284 | 133494018 |
| **2017** | 3736 | 1172 | 2564 | 2767 | 430 | 143 | 30 | 350 | 135229289 |
| **2018** | 4130 | 1314 | 2816 | 3059 | 482 | 193 | 37 | 345 | 136335528 |
| **2019** | 4507 | 1439 | 3068 | 3300 | 551 | 194 | 42 | 413 | 137381702 |
| **2020** | 4437 | 1378 | 3059 | 3299 | 489 | 202 | 30 | 412 | 138429175 |
| **2021** | 4907 | 1509 | 3398 | 3564 | 555 | 221 | 41 | 451 | 139339453 |
| **2022** | 5297 | 1626 | 3671 | 3742 | 637 | 257 | 31 | 561 | 140311934 |
| **2023** | 5755 | 1797 | 3958 | 4138 | 661 | 242 | 50 | 585 | 141596553 |
| **Total** | 78903 | 27823 | 51080 | 61578 | 7620 | 2753 | 518 | 6028 | 3043725672 |

**Supplementary Table 2 Overall and Sex‐Stratified Cardiogenic Shock and Chronic Ischemic Heart Disease –related Age-Adjusted Mortality Rates per 1,000,000 in Adults in the United States 1999-2023**

| **Age Adjusted Rate (95% CI)** | | | |
| --- | --- | --- | --- |
| **Year** | **Men** | **Women** | **Overall** |
| **1999** | 49.68 (47.4 to 51.97 ) | 26.33 (25.01 to 27.66 ) | 35.88 (34.68 to 37.09 ) |
| **2000** | 48.27 (46.04 to 50.5 ) | 23.48 (22.23 to 24.73 ) | 33.61 (32.45 to 34.77 ) |
| **2001** | 41.51 (39.46 to 43.55 ) | 19.72 (18.58 to 20.86 ) | 28.52 (27.46 to 29.58 ) |
| **2002** | 36.83 (34.93 to 38.74 ) | 18.11 (17.02 to 19.19 ) | 25.84 (24.84 to 26.84 ) |
| **2003** | 34.14 (32.32 to 35.95 ) | 15.67 (14.67 to 16.67 ) | 23.33 (22.39 to 24.27 ) |
| **2004** | 33.18 (31.4 to 34.96 ) | 15.77 (14.77 to 16.77 ) | 22.87 (21.94 to 23.8 ) |
| **2005** | 30.08 (28.41 to 31.75 ) | 13.72 (12.8 to 14.65 ) | 20.45 (19.59 to 21.32 ) |
| **2006** | 29.72 (28.07 to 31.36 ) | 13.7 (12.78 to 14.62 ) | 20.21 (19.35 to 21.06 ) |
| **2007** | 27.6 (26.04 to 29.15 ) | 13.04 (12.15 to 13.93 ) | 19.18 (18.35 to 20 ) |
| **2008** | 27.65 (26.11 to 29.18 ) | 13.26 (12.36 to 14.15 ) | 19.31 (18.49 to 20.13 ) |
| **2009** | 27.72 (26.19 to 29.24 ) | 11.89 (11.06 to 12.73 ) | 18.49 (17.7 to 19.29 ) |
| **2010** | 28.82 (27.28 to 30.36 ) | 12.4 (11.55 to 13.25 ) | 19.37 (18.56 to 20.18 ) |
| **2011** | 29.45 (27.93 to 30.97 ) | 12.18 (11.34 to 13.01 ) | 19.61 (18.81 to 20.41 ) |
| **2012** | 29.46 (27.97 to 30.96 ) | 10.6 (9.83 to 11.36 ) | 18.74 (17.97 to 19.52 ) |
| **2013** | 31.02 (29.5 to 32.53 ) | 11.41 (10.62 to 12.2 ) | 19.9 (19.11 to 20.69 ) |
| **2014** | 33.61 (32.06 to 35.16 ) | 12.44 (11.62 to 13.26 ) | 21.7 (20.88 to 22.51 ) |
| **2015** | 38.43 (36.8 to 40.06 ) | 14.45 (13.57 to 15.33 ) | 24.91 (24.05 to 25.77 ) |
| **2016** | 38.61 (36.99 to 40.22 ) | 14.48 (13.61 to 15.34 ) | 25.14 (24.28 to 26 ) |
| **2017** | 41.85 (40.2 to 43.51 ) | 15.21 (14.33 to 16.09 ) | 26.99 (26.11 to 27.87 ) |
| **2018** | 44.91 (43.22 to 46.6 ) | 16.77 (15.85 to 17.69 ) | 29.23 (28.33 to 30.14 ) |
| **2019** | 47.58 (45.87 to 49.3 ) | 17.82 (16.88 to 18.75 ) | 31.02 (30.1 to 31.93 ) |
| **2020** | 46.52 (44.84 to 48.19 ) | 16.83 (15.93 to 17.73 ) | 30.06 (29.17 to 30.96 ) |
| **Total** | 36.41 (36.05 to 36.77 ) | 15.31 (15.11 to 15.51 ) | 24.35 (24.16 to 24.54 ) |
| **2021** | 51.65 (49.87 to 53.43 ) | 18.89 (17.93 to 19.86 ) | 33.53 (32.58 to 34.48 ) |
| **2022** | 54.57 (52.77 to 56.37 ) | 19.43 (18.47 to 20.38 ) | 34.96 (34.01 to 35.92 ) |
| **2023** | 57.45 (55.63 to 59.28 ) | 21.26 (20.27 to 22.26 ) | 37.44 (36.46 to 38.42 ) |
| **Total** | 54.61 (53.57 to 55.65 ) | 19.88 (19.32 to 20.45 ) | 35.33 (34.78 to 35.89 ) |

**Supplemental Table 3 Annual Percent Change (APC) and Average Annual Percent Change (AAPC) of Cardiogenic Shock and Chronic Ischemic Heart Disease –related Age-Adjusted Mortality Rates per 1,000,000 in Adults in the United States 1999-2023**

| **Year Interval** | **APC (95% CI)** | **Year Interval** | **AAPC (95% CI)** |
| --- | --- | --- | --- |
| **Overall** |  |  |  |
| **1999-2005** | -9.2074*( -13.495 to -6.055 ) | 1999-2023 | 0.1598( -0.1807 to 0.5667 ) |
| **2005-2012** | -0.9879( -10.9482 to 2.2114 ) |  |  |
| **2012-2015** | 9.2648( -1.9112 to 12.6148 ) |  |  |
| **2015-2023** | 5.4108*( 1.8768 to 6.7627 ) |  |  |
| **Females** |  |  |  |
| **1999-2003** | -12.4785*( -18.5328 to -9.1134 ) | 1999-2023 | -0.9069*( -1.2251 to -0.4983 ) |
| **2003-2012** | -3.4389*( -5.2694 to -0.9923 ) |  |  |
| **2012-2023** | 5.8891*( 4.9944 to 7.0771 ) |  |  |
| **Males** |  |  |  |
| **1999-2003** | -9.8645*( -15.664 to -6.1105 ) | 1999-2023 | 0.4983*( 0.2105 to 0.9981 ) |
| **2003-2009** | -4.0891( -6.5373 to 9.0143 ) |  |  |
| **2009-2023** | 5.7689*( 5.0359 to 7.3163 ) |  |  |
| **American Indian or Alaska Native** |  |  |  |
| **2014-2023** | 1.3462( -6.2008 to 9.7972 ) | 2014-2023 | 1.3462( -6.2008 to 9.7972 ) |
| **Asian or Pacific Islander** |  |  |  |
| **1999-2012** | -2.8944*( -9.4651 to -0.0665 ) | 1999-2020 | 0.9914( -0.2918 to 2.5803 ) |
| **2012-2020** | 7.6402*( 3.9128 to 19.8196 ) |  |  |
| **2021-2023** | 0.5185( -7.7354 to 9.7996 ) |  |  |
| **Black or African American** |  |  |  |
| **1999-2006** | -8.9846*( -14.6425 to -5.5521 ) | 1999-2023 | 1.8570*( 1.2601 to 2.6242 ) |
| **2006-2023** | 6.6882*( 5.9264 to 7.8505 ) |  |  |
| **White - 2 Joinpoints** |  |  |  |
| **1999-2005** | -9.4724*( -13.4596 to -7.6519 ) | 1999-2023 | 0.0284( -0.2603 to 0.3825 ) |
| **2005-2012** | -0.5857( -4.2841 to 2.9836 ) |  |  |
| **2012-2023** | 6.0392*( 5.2801 to 7.414 ) |  |  |
| **Hispanic or Latino -** |  |  |  |
| **1999-2004** | -10.7746*( -20.6925 to -5.6418 ) | 1999-2023 | 0.2104( -0.3517 to 1.0408 ) |
| **2004-2012** | -1.4824( -5.1183 to 6.0906 ) |  |  |
| **2012-2023** | 6.9581*( 5.6923 to 9.8636 ) |  |  |
| **Census Region 1: Northeast** |  |  |  |
| **1999-2003** | -10.5168*( -16.2085 to -7.1575 ) | 1999-2023 | 0.059( -0.296 to 0.4704 ) |
| **2003-2010** | -3.8682( -5.9409 to 3.9178 ) |  |  |
| **2010-2023** | 5.8150*( 5.1006 to 6.8979 ) |  |  |
| **Census Region 2: Midwest** |  |  |  |
| **1999-2005** | -10.1792*( -14.9571 to -7.8954 ) | 1999-2023 | -0.0067( -0.3882 to 0.4359 ) |
| **2005-2013** | 1.1757( -4.8156 to 3.5474 ) |  |  |
| **2013-2023** | 5.6435*( 4.5337 to 9.1249 ) |  |  |
| **Census Region 3: South** |  |  |  |
| **1999-2006** | -9.0767*( -11.804 to -7.6736 ) | 1999-2023 | 0.0557( -0.2757 to 0.3648 ) |
| **2006-2012** | -1.1043( -4.7888 to 2.8157 ) |  |  |
| **2012-2017** | 10.3053*( 7.8668 to 15.6616 ) |  |  |
| **2017-2023** | 4.3531*( 2.0261 to 5.6179 ) |  |  |
| **Census Region 4: West** |  |  |  |
| **1999-2003** | -10.8271*( -17.5265 to -7.1355 ) | 1999-2023 | 0.3214( -0.0151 to 0.7623 ) |
| **2003-2012** | -1.2017( -3.1239 to 1.1237 ) |  |  |
| **2012-2023** | 6.0312*( 5.2389 to 7.1737 ) |  |  |
| **metro** |  |  |  |
| **1999-2005** | -9.1738*( -12.9344 to -7.0387 ) | 1999-2020 | -0.7817*( -1.1842 to -0.3453 ) |
| **2005-2012** | -1.0014( -6.7456 to 2.0193 ) |  |  |
| **2012-2018** | 8.0714( -0.7563 to 13.2461 ) |  |  |
| **2018-2020** | 0.866( -3.5821 to 6.6001 ) |  |  |
| **non metro** |  |  |  |
| **1999-2007** | -8.6043*( -10.811 to -6.924 ) | 1999-2020 | -0.5496*( -0.9758 to -0.053 ) |
| **2007-2020** | 4.7562*( 3.8907 to 5.9092 ) |  |  |
| **45 to 64** |  |  |  |
| **1999-2005** | -8.8710*( -11.0157 to -7.5022 ) | 1999-2023 | 1.6790*( 1.4256 to 1.9417 ) |
| **2005-2010** | 0.651( -2.675 to 4.2546 ) |  |  |
| **2010-2018** | 9.2508*( 8.3498 to 11.47 ) |  |  |
| **2018-2023** | 4.4297*( 2.6307 to 5.6841 ) |  |  |
| **65+** |  |  |  |
| **1999-2005** | -9.3640*( -12.5966 to -7.6364 ) | 1999-2023 | -0.1232( -0.3959 to 0.1951 ) |
| **2005-2012** | -1.0171( -3.9432 to 2.1264 ) |  |  |
| **2012-2023** | 5.9125*( 5.2102 to 7.0146 ) |  |  |

**Supplementary Table 4** **Cardiogenic Shock and Chronic Ischemic Heart Disease -related Age-Adjusted Mortality Rates per 1,000,000, Stratified by Race in Adults in the United States 1999-2023**

| **Age Adjusted Rate (95% CI)** | | | | | |
| --- | --- | --- | --- | --- | --- |
| **Year** | **NH White** | **NH Black or African American** | **NH American Indian or Alaska Native** | **Hispanic or Latino** | **NH Asian or Pacific Islander** |
| **1999** | 36.82 (35.5 to 38.15 ) | 28.43 (24.67 to 32.19 ) | Unreliable | 34.05 (28.46 to 39.63 ) | 25.24 (18.8 to 33.19 ) |
| **2000** | 34.36 (33.08 to 35.63 ) | 26.35 (22.77 to 29.92 ) | Unreliable | 32.46 (27.1 to 37.81 ) | 24.67 (18.68 to 31.96 ) |
| **2001** | 29.01 (27.84 to 30.18 ) | 24.28 (20.85 to 27.71 ) | Unreliable | 31.04 (25.99 to 36.1 ) | 21.53 (16.03 to 28.31 ) |
| **2002** | 26.46 (25.35 to 27.57 ) | 21.04 (17.88 to 24.2 ) | Unreliable | 25.87 (21.37 to 30.36 ) | 20.66 (15.18 to 27.47 ) |
| **2003** | 24.13 (23.07 to 25.18 ) | 17.62 (14.77 to 20.48 ) | Unreliable | 21.09 (17.15 to 25.03 ) | 17.58 (12.87 to 23.45 ) |
| **2004** | 23.54 (22.51 to 24.58 ) | 19.5 (16.5 to 22.51 ) | Unreliable | 19.51 (15.81 to 23.21 ) | 19.82 (14.8 to 25.99 ) |
| **2005** | 20.62 (19.66 to 21.58 ) | 17.11 (14.33 to 19.89 ) | Unreliable | 20.38 (16.7 to 24.06 ) | 22.78 (17.76 to 28.78 ) |
| **2006** | 20.71 (19.75 to 21.67 ) | 15.37 (12.8 to 17.93 ) | Unreliable | 19.54 (16.02 to 23.07 ) | 16.53 (12.27 to 21.79 ) |
| **2007** | 19.23 (18.32 to 20.15 ) | 17.55 (14.82 to 20.29 ) | Unreliable | 21.74 (18.18 to 25.31 ) | 12.67 (9.17 to 17.06 ) |
| **2008** | 19.51 (18.59 to 20.43 ) | 16.8 (14.2 to 19.41 ) | Unreliable | 18.97 (15.68 to 22.25 ) | 19.78 (15.39 to 25.04 ) |
| **2009** | 18.9 (18 to 19.79 ) | 15.4 (12.89 to 17.91 ) | Unreliable | 17.72 (14.63 to 20.81 ) | 13.8 (10.34 to 18.05 ) |
| **2010** | 19.51 (18.6 to 20.41 ) | 17.94 (15.27 to 20.6 ) | Unreliable | 16.97 (14.05 to 19.89 ) | 20.91 (16.6 to 25.99 ) |
| **2011** | 19.61 (18.71 to 20.51 ) | 21.15 (18.36 to 23.94 ) | Unreliable | 18.34 (15.44 to 21.24 ) | 15.06 (11.62 to 19.19 ) |
| **2012** | 18.98 (18.1 to 19.85 ) | 18.33 (15.77 to 20.89 ) | Unreliable | 17.38 (14.62 to 20.14 ) | 14.12 (10.94 to 17.94 ) |
| **2013** | 19.86 (18.97 to 20.76 ) | 22.75 (19.92 to 25.57 ) | Unreliable | 16.77 (14.14 to 19.41 ) | 17.33 (13.84 to 21.43 ) |
| **2014** | 21.29 (20.37 to 22.21 ) | 23.27 (20.49 to 26.05 ) | 31.16 (19.29 to 47.63 ) | 21.73 (18.81 to 24.64 ) | 20.64 (16.79 to 24.49 ) |
| **2015** | 24.52 (23.54 to 25.5 ) | 28.36 (25.35 to 31.36 ) | 51.54 (36.29 to 71.04 ) | 23.51 (20.58 to 26.45 ) | 20.98 (17.2 to 24.76 ) |
| **2016** | 25.04 (24.06 to 26.02 ) | 27.57 (24.64 to 30.51 ) | 37 (24.38 to 53.83 ) | 24.09 (21.19 to 26.99 ) | 19.9 (16.34 to 23.46 ) |
| **2017** | 26.31 (25.32 to 27.31 ) | 31.87 (28.76 to 34.98 ) | 31.41 (20.87 to 45.4 ) | 27.84 (24.82 to 30.86 ) | 21.26 (17.71 to 24.8 ) |
| **2018** | 28.66 (27.63 to 29.69 ) | 35.03 (31.81 to 38.25 ) | 42.21 (29.4 to 58.71 ) | 26.04 (23.19 to 28.88 ) | 27.7 (23.73 to 31.67 ) |
| **2019** | 30.22 (29.18 to 31.27 ) | 38.01 (34.74 to 41.28 ) | 40.71 (28.95 to 55.65 ) | 30.43 (27.4 to 33.46 ) | 27.02 (23.16 to 30.88 ) |
| **2020** | 29.95 (28.91 to 30.99 ) | 33.24 (30.2 to 36.27 ) | 29.95 (19.9 to 43.28 ) | 28.63 (25.77 to 31.49 ) | 26.55 (22.84 to 30.27 ) |
| **Total** | 24.36 (24.16 to 24.56 ) | 24.34 (23.7 to 24.98 ) | 30.13 (27.14 to 33.12 ) | 23.34 (22.63 to 24.05 ) | 20.56 (19.64 to 21.47 ) |
| **2021** | 33.12 (32.01 to 34.22 ) | 37.64 (34.4 to 40.88 ) | 44.71 (31.64 to 61.37 ) | 31.02 (28.04 to 33.99 ) | 29.81 (25.82 to 33.8 ) |
| **2022** | 33.64 (32.54 to 34.73 ) | 42.52 (39.12 to 45.91 ) | 30.27 (20.43 to 43.22 ) | 36.77 (33.62 to 39.91 ) | 33.79 (29.61 to 37.96 ) |
| **2023** | 36.88 (35.73 to 38.02 ) | 43.12 (39.74 to 46.5 ) | 53.04 (39.11 to 70.32 ) | 37.45 (34.31 to 40.59 ) | 30.26 (26.41 to 34.11 ) |
| **Total** | 34.55 (33.91 to 35.2 ) | 41.15 (39.22 to 43.09 ) | 42.95 (35.15 to 50.76 ) | 35.21 (33.43 to 37 ) | 31.31 (28.99 to 33.62 ) |

**Supplementary Table 5 Cardiogenic Shock and Chronic Ischemic Heart Disease -related Age-Adjusted Mortality Rate per 1,000,000 Stratified by Census Region in Adults in the United States 1999-2023**

| Age Adjusted Rate (95% CI) | | | | |
| --- | --- | --- | --- | --- |
| **Year** | **Northeast** | **Midwest** | **South** | **West** |
| **1999** | 36.41 ( 33.78 to 39.05 ) | 34.75 ( 32.33 to 37.17 ) | 35.41 ( 33.39 to 37.43 ) | 37.41 ( 34.64 to 40.18 ) |
| **2000** | 33.51 ( 30.99 to 36.02 ) | 32.67 ( 30.33 to 35.01 ) | 33.61 ( 31.65 to 35.56 ) | 34.72 ( 32.07 to 37.36 ) |
| **2001** | 27.26 ( 25 to 29.51 ) | 26.27 ( 24.18 to 28.35 ) | 29.86 ( 28.04 to 31.68 ) | 29.92 ( 27.5 to 32.34 ) |
| **2002** | 26.11 ( 23.91 to 28.31 ) | 24.43 ( 22.43 to 26.44 ) | 25.32 ( 23.66 to 26.99 ) | 27.9 ( 25.59 to 30.22 ) |
| **2003** | 23.94 ( 21.85 to 26.02 ) | 22.95 ( 21.02 to 24.89 ) | 22.79 ( 21.22 to 24.36 ) | 24.15 ( 22.02 to 26.28 ) |
| **2004** | 22.28 ( 20.26 to 24.29 ) | 20.7 ( 18.87 to 22.52 ) | 23.29 ( 21.73 to 24.86 ) | 24.98 ( 22.83 to 27.13 ) |
| **2005** | 20.74 ( 18.8 to 22.67 ) | 17.72 ( 16.04 to 19.39 ) | 20.08 ( 18.64 to 21.52 ) | 23.75 ( 21.69 to 25.81 ) |
| **2006** | 20.72 ( 18.79 to 22.64 ) | 19.38 ( 17.63 to 21.12 ) | 19.03 ( 17.64 to 20.41 ) | 22.54 ( 20.55 to 24.52 ) |
| **2007** | 21.65 ( 19.69 to 23.61 ) | 17.43 ( 15.79 to 19.07 ) | 17.72 ( 16.39 to 19.04 ) | 20.97 ( 19.08 to 22.86 ) |
| **2008** | 18.72 ( 16.91 to 20.53 ) | 19.05 ( 17.35 to 20.75 ) | 18.36 ( 17.03 to 19.69 ) | 21.43 ( 19.54 to 23.32 ) |
| **2009** | 16.95 ( 15.23 to 18.67 ) | 19.48 ( 17.77 to 21.19 ) | 16.96 ( 15.7 to 18.23 ) | 21.44 ( 19.58 to 23.3 ) |
| **2010** | 18.48 ( 16.7 to 20.27 ) | 19.61 ( 17.9 to 21.32 ) | 17.24 ( 15.98 to 18.5 ) | 23.43 ( 21.49 to 25.37 ) |
| **2011** | 18.37 ( 16.6 to 20.13 ) | 20.85 ( 19.1 to 22.6 ) | 17.57 ( 16.32 to 18.83 ) | 22.82 ( 20.95 to 24.7 ) |
| **2012** | 18.23 ( 16.49 to 19.97 ) | 18.83 ( 17.18 to 20.47 ) | 17.33 ( 16.1 to 18.56 ) | 21.35 ( 19.57 to 23.12 ) |
| **2013** | 20.47 ( 18.63 to 22.31 ) | 19.19 ( 17.55 to 20.84 ) | 18.94 ( 17.67 to 20.2 ) | 21.63 ( 19.86 to 23.4 ) |
| **2014** | 23.25 ( 21.31 to 25.18 ) | 20.48 ( 18.78 to 22.17 ) | 20.19 ( 18.91 to 21.48 ) | 23.97 ( 22.14 to 25.81 ) |
| **2015** | 24.07 ( 22.11 to 26.03 ) | 24.92 ( 23.08 to 26.75 ) | 24.13 ( 22.74 to 25.53 ) | 26.77 ( 24.87 to 28.68 ) |
| **2016** | 24.97 ( 22.98 to 26.96 ) | 22.39 ( 20.65 to 24.13 ) | 24.55 ( 23.17 to 25.92 ) | 28.73 ( 26.78 to 30.68 ) |
| **2017** | 25.19 ( 23.23 to 27.16 ) | 24.54 ( 22.73 to 26.34 ) | 27.92 ( 26.47 to 29.37 ) | 29.24 ( 27.29 to 31.18 ) |
| **2018** | 30.5 ( 28.33 to 32.67 ) | 25.52 ( 23.7 to 27.34 ) | 29.43 ( 27.96 to 30.9 ) | 31.43 ( 29.45 to 33.4 ) |
| **2019** | 28.94 ( 26.87 to 31.01 ) | 28.65 ( 26.75 to 30.56 ) | 32.31 ( 30.79 to 33.83 ) | 32.69 ( 30.7 to 34.68 ) |
| **2020** | 30.92 ( 28.8 to 33.05 ) | 29 ( 27.09 to 30.91 ) | 29.19 ( 27.77 to 30.62 ) | 31.64 ( 29.7 to 33.57 ) |
| **Total** | 24.21 ( 23.78 to 24.64 ) | 29 ( 27.09 to 30.91 ) | 23.74 ( 23.43 to 24.05 ) | 26.55 ( 26.12 to 26.98 ) |
| **2021** | 33.53 ( 31.31 to 35.75 ) | 31.75 ( 29.72 to 33.77 ) | 32.85 ( 31.32 to 34.38 ) | 36.32 ( 34.23 to 38.42 ) |
| **2022** | 32.46 ( 30.32 to 34.6 ) | 31.85 ( 29.86 to 33.84 ) | 34.82 ( 33.28 to 36.36 ) | 40.14 ( 37.99 to 42.28 ) |
| **2023** | 37.44 ( 35.15 to 39.74 ) | 35.16 ( 33.09 to 37.23 ) | 36.63 ( 35.07 to 38.2 ) | 40.8 ( 38.64 to 42.95 ) |
| **Total** | 34.53 ( 33.24 to 35.81 ) | 32.94 ( 31.77 to 34.11 ) | 34.81 ( 33.92 to 35.7 ) | 39.09 ( 37.86 to 40.32 ) |

**Supplementary Table 6 Cardiogenic Shock and Chronic Ischemic Heart Disease –related Age-Adjusted Mortality Rates per 1,000,000, Stratified by States in Adults in the United States 1999-2020**

| **State** | **Age Adjusted Rate (95% CI)** |
| --- | --- |
| Alabama | 22.48 (21.03 to 23.93 ) |
| Alaska | 17.26 (13.17 to 22.22 ) |
| Arizona | 31.23 (29.74 to 32.71 ) |
| Arkansas | 29.32 (27.22 to 31.41 ) |
| California | 29.4 (28.76 to 30.04 ) |
| Colorado | 15.4 (14.1 to 16.69 ) |
| Connecticut | 25.68 (23.94 to 27.42 ) |
| Delaware | 20.35 (17.2 to 23.5 ) |
| District of Columbia | 37.61 (31.97 to 43.26 ) |
| Florida | 22.23 (21.58 to 22.88 ) |
| Georgia | 25.49 (24.27 to 26.72 ) |
| Hawaii | 24.69 (21.9 to 27.48 ) |
| Idaho | 19.04 (16.58 to 21.5 ) |
| Illinois | 18.51 (17.68 to 19.33 ) |
| Indiana | 25.79 (24.43 to 27.16 ) |
| Iowa | 24.89 (23.06 to 26.71 ) |
| Kansas | 23.07 (21.16 to 24.98 ) |
| Kentucky | 22.42 (20.88 to 23.97 ) |
| Louisiana | 23.27 (21.69 to 24.84 ) |
| Maine | 23.55 (20.94 to 26.15 ) |
| Maryland | 21.94 (20.57 to 23.3 ) |
| Massachusetts | 23.23 (21.99 to 24.46 ) |
| Michigan | 24.45 (23.41 to 25.49 ) |
| Minnesota | 13.99 (12.88 to 15.1 ) |
| Mississippi | 28.21 (26.08 to 30.34 ) |
| Missouri | 26.26 (24.88 to 27.64 ) |
| Montana | 14.84 (12.36 to 17.33 ) |
| Nebraska | 28.5 (25.89 to 31.11 ) |
| Nevada | 31.6 (29.1 to 34.1 ) |
| New Hampshire | 25.44 (22.51 to 28.37 ) |
| New Jersey | 21.91 (20.86 to 22.97 ) |
| New Mexico | 17.62 (15.61 to 19.63 ) |
| New York | 22.4 (21.68 to 23.11 ) |
| North Carolina | 22.66 (21.6 to 23.72 ) |
| North Dakota | 34.17 (29.74 to 38.6 ) |
| Ohio | 29.24 (28.19 to 30.28 ) |
| Oklahoma | 23.96 (22.25 to 25.68 ) |
| Oregon | 18.15 (16.72 to 19.59 ) |
| Pennsylvania | 27.63 (26.71 to 28.55 ) |
| Rhode Island | 28.72 (25.4 to 32.04 ) |
| South Carolina | 24.98 (23.4 to 26.55 ) |
| South Dakota | 20.73 (17.49 to 23.96 ) |
| Tennessee | 25.24 (23.88 to 26.59 ) |
| Texas | 26.36 (25.58 to 27.14 ) |
| Utah | 17.69 (15.61 to 19.77 ) |
| Vermont | 24.57 (20.5 to 28.64 ) |
| Virginia | 16.67 (15.65 to 17.69 ) |
| Washington | 28.89 (27.44 to 30.34 ) |
| West Virginia | 29.08 (26.61 to 31.55 ) |
| Wisconsin | 16.04 (14.93 to 17.15 ) |
| Wyoming | 20.18 (16.2 to 24.83 ) |
| Total | 24.35 (24.16 to 24.54 ) |

**Supplementary Table 7 Cardiogenic Shock and Chronic Ischemic Heart Disease–related Age-Adjusted Mortality Rates per 1,000,000, Stratified by States in Adults in the United States 1999-2020 ranked according to Percentiles.**

| **State** | **Age Adjusted Rate** | **Percentile** | **Rank** |
| --- | --- | --- | --- |
| District of Columbia | 37.61 | 100 | 1 |
| North Dakota | 34.17 | 98 | 2 |
| Nevada | 31.6 | 96 | 3 |
| Arizona | 31.23 | 94 | 4 |
| California | 29.4 | 92 | 5 |
| Arkansas | 29.32 | 90 | 6 |
| Ohio | 29.24 | 88 | 7 |
| West Virginia | 29.08 | 86 | 8 |
| Washington | 28.89 | 84 | 9 |
| Rhode Island | 28.72 | 82 | 10 |
| Nebraska | 28.5 | 80 | 11 |
| Mississippi | 28.21 | 78 | 12 |
| Pennsylvania | 27.63 | 76 | 13 |
| Texas | 26.36 | 74 | 14 |
| Missouri | 26.26 | 72 | 15 |
| Indiana | 25.79 | 70 | 16 |
| Connecticut | 25.68 | 68 | 17 |
| Georgia | 25.49 | 66 | 18 |
| New Hampshire | 25.44 | 64 | 19 |
| Tennessee | 25.24 | 62 | 20 |
| South Carolina | 24.98 | 60 | 21 |
| Iowa | 24.89 | 58 | 22 |
| Hawaii | 24.69 | 56 | 23 |
| Vermont | 24.57 | 54 | 24 |
| Michigan | 24.45 | 52 | 25 |
| Oklahoma | 23.96 | 50 | 26 |
| Maine | 23.55 | 48 | 27 |
| Louisiana | 23.27 | 46 | 28 |
| Massachusetts | 23.23 | 44 | 29 |
| Kansas | 23.07 | 42 | 30 |
| North Carolina | 22.66 | 40 | 31 |
| Alabama | 22.48 | 38 | 32 |
| Kentucky | 22.42 | 36 | 33 |
| New York | 22.4 | 34 | 34 |
| Florida | 22.23 | 32 | 35 |
| Maryland | 21.94 | 30 | 36 |
| New Jersey | 21.91 | 28 | 37 |
| South Dakota | 20.73 | 26 | 38 |
| Delaware | 20.35 | 24 | 39 |
| Wyoming | 20.18 | 22 | 40 |
| Idaho | 19.04 | 20 | 41 |
| Illinois | 18.51 | 18 | 42 |
| Oregon | 18.15 | 16 | 43 |
| Utah | 17.69 | 14 | 44 |
| New Mexico | 17.62 | 12 | 45 |
| Alaska | 17.26 | 10 | 46 |
| Virginia | 16.67 | 8 | 47 |
| Wisconsin | 16.04 | 6 | 48 |
| Colorado | 15.4 | 4 | 49 |
| Montana | 14.84 | 2 | 50 |
| Minnesota | 13.99 | 0 | 51 |

**Supplementary Table 8 Cardiogenic Shock and Chronic Ischemic Heart Disease -related Age-Adjusted Mortality Rates per 1,000,000 in the Metropolitan and Non-metropolitan areas in Adults in the United States 1999-2020**

| **Age Adjusted Mortality (95% CI)** | | |
| --- | --- | --- |
| **Year** | **Metro** | **Non-Metro** |
| **1999** | 35.19 (33.87 to 36.52 ) | 38.69 (35.83 to 41.56 ) |
| **2000** | 33.84 (32.55 to 35.13 ) | 32.59 (29.97 to 35.21 ) |
| **2001** | 27.67 (26.51 to 28.83 ) | 32.33 (29.73 to 34.93 ) |
| **2002** | 25.24 (24.14 to 26.34 ) | 28.27 (25.85 to 30.68 ) |
| **2003** | 23.24 (22.19 to 24.28 ) | 24 (21.78 to 26.22 ) |
| **2004** | 22.45 (21.43 to 23.47 ) | 24.92 (22.67 to 27.16 ) |
| **2005** | 20.4 (19.44 to 21.36 ) | 20.82 (18.77 to 22.86 ) |
| **2006** | 19.81 (18.87 to 20.75 ) | 22 (19.92 to 24.09 ) |
| **2007** | 19.3 (18.39 to 20.22 ) | 18.62 (16.72 to 20.51 ) |
| **2008** | 18.96 (18.06 to 19.86 ) | 20.91 (18.9 to 22.91 ) |
| **2009** | 18.26 (17.38 to 19.13 ) | 19.58 (17.65 to 21.51 ) |
| **2010** | 18.91 (18.03 to 19.8 ) | 21.42 (19.42 to 23.43 ) |
| **2011** | 19.26 (18.38 to 20.13 ) | 21.46 (19.47 to 23.45 ) |
| **2012** | 18.47 (17.62 to 19.32 ) | 20.22 (18.3 to 22.13 ) |
| **2013** | 19.41 (18.55 to 20.27 ) | 22.3 (20.3 to 24.3 ) |
| **2014** | 21.32 (20.43 to 22.21 ) | 23.47 (21.43 to 25.5 ) |
| **2015** | 24.33 (23.39 to 25.26 ) | 27.73 (25.52 to 29.94 ) |
| **2016** | 24.56 (23.63 to 25.49 ) | 28.12 (25.92 to 30.33 ) |
| **2017** | 26.57 (25.62 to 27.53 ) | 29.28 (27.05 to 31.51 ) |
| **2018** | 29.26 (28.27 to 30.25 ) | 29.38 (27.15 to 31.6 ) |
| **2019** | 30.56 (29.56 to 31.56 ) | 33.35 (31.02 to 35.67 ) |
| **2020** | 29.37 (28.4 to 30.34 ) | 33.75 (31.4 to 36.1 ) |
| **Total** | 23.99 (23.78 to 24.2 ) | 26.09 (25.62 to 26.56 ) |

**Supplementary Table 9 Cardiogenic Shock and Chronic Ischemic Heart Disease-related Mortality, Stratified by Place of Death in Adults in the United States 1999-2023**

| **Year** | **Medical Facility - Inpatient** | **Decedent's home** | **Nursing home/long term care** | **Other** | **Hospice facility** |
| --- | --- | --- | --- | --- | --- |
| **1999** | 2716 | 141 | 334 | 19 | Missing |
| **2000** | 2583 | 123 | 298 | 30 | Missing |
| **2001** | 2317 | 109 | 186 | 28 | Missing |
| **2002** | 2078 | 111 | 187 | 25 | Missing |
| **2003** | 1898 | 116 | 168 | 14 | Suppressed |
| **2004** | 1905 | 121 | 154 | 15 | Suppressed |
| **2005** | 1759 | 91 | 130 | 24 | Suppressed |
| **2006** | 1774 | 93 | 136 | 17 | Suppressed |
| **2007** | 1662 | 98 | 146 | 14 | Suppressed |
| **2008** | 1711 | 109 | 154 | 16 | Suppressed |
| **2009** | 1663 | 106 | 124 | 13 | 19 |
| **2010** | 1811 | 122 | 103 | 18 | 43 |
| **2011** | 1910 | 127 | 102 | 20 | 39 |
| **2012** | 1882 | 115 | 81 | 21 | 55 |
| **2013** | 2072 | 116 | 89 | 16 | 41 |
| **2014** | 2334 | 123 | 104 | 27 | 39 |
| **2015** | 2812 | 150 | 98 | 21 | 57 |
| **2016** | 2947 | 147 | 80 | 29 | 52 |
| **2017** | 3303 | 111 | 71 | 25 | 65 |
| **2018** | 3686 | 120 | 64 | 19 | 74 |
| **2019** | 3990 | 150 | 83 | 35 | 96 |
| **2020** | 3925 | 151 | 88 | 29 | 89 |
| **2021** | 4366 | 145 | 54 | 39 | 121 |
| **2022** | 4756 | 132 | 73 | 32 | 106 |
| **2023** | 5187 | 145 | 64 | 35 | 124 |
| **Total** | 67047 | 3072 | 3171 | 581 | 1020 |
